# The Effects of Low- and High-Glycemic Index Sport Nutrition Bars on Metabolism and Performance in Recreational Soccer Players

**DOI:** 10.3390/nu12040982

**Published:** 2020-04-02

**Authors:** Mojtaba Kaviani, Philip D. Chilibeck, Spencer Gall, Jennifer Jochim, Gordon A. Zello

**Affiliations:** 1 School of Nutrition and Dietetics, Faculty of Pure & Applied Science, Acadia University, Wolfville, Nova Scotia, NS B4P 2R6, Canada; 2 College of Kinesiology, University of Saskatchewan, 87 Campus Dr, Saskatoon, SK S7N 5B2, Canada; Spencer.Gall@usask.ca (S.G.); Jennifer.Jochim@usask.ca (J.J.); 3 College of Pharmacy and Nutrition, University of Saskatchewan, 107 Wiggins Rd, Saskatoon, SK S7N 5E5, Canada; gordon.zello@usask.ca

**Keywords:** Carbohydrate, high-intensity exercise, fatigue

## Abstract

Consumption of low-glycemic index (GI) carbohydrates (CHO) may be superior to high-GI CHO before exercise by increasing fat oxidation and decreasing carbohydrate oxidation. We compared the effects of pre-exercise feeding of a low-GI lentil-based sports nutrition bar with a high-GI bar on metabolism and performance during a simulated soccer match. Using a randomized, double-blind, counterbalanced, crossover design, participants (*n* = 8) consumed 1.5 g/kg available CHO from a low-GI bar (GI = 45) or high-GI bar (GI = 101) two hours before a 90 min simulated soccer match, and 0.38 g/kg body mass during a 15 min half-time break. The test involved alternating 6 min intervals of paced jogging, running, walking, and sprinting, and 3 min intervals of soccer-specific skills (timed ball dribbling, agility running, heading, kicking accuracy). Carbohydrate oxidation rate was lower during the match after consuming the low-GI compared to high-GI bar (2.17 ± 0.6 vs. 2.72 ± 0.4 g/min; *p* < 0.05). Participants performed better during the low-GI versus high-GI bar condition on the agility test (5.7 ± 0.4 versus 6.1 ± 0.6 s; *p* < 0.01) and heading (i.e., jumping height 24.7 ± 4.3 versus 22.2 ± 4.5 cm; *p* < 0.01) late in the soccer match (72 min). A low-GI lentil-based sports nutrition bar provides a metabolic benefit (lower carbohydrate oxidation rate) and a modest improvement in agility running and jumping height (heading) late in the test.

## 1. Introduction

Carbohydrate (CHO) is an important source of energy throughout strenuous prolonged exercise. Premature fatigue during prolonged exercise is linked with depletion of carbohydrate stores (i.e., blood glucose and liver and muscle glycogen stores). Thus, carbohydrate consumption before and during exercise improves exercise performance compared with a fasted condition [1,2]. Muscle glycogen concentrations are directly correlated to time to fatigue during moderately strenuous exercise ranging from 60%–80% of maximal oxygen uptake (VO_2max_) [1]. Thus, endurance and high-intensity intermittent exercise will be adversely affected by reduced glycogen stores. During soccer matches, this would most likely occur in the second half of a game [3,4,5]. Soccer players with lower levels of muscle glycogen cover less distance and run at lower speeds during the last 15 min of a match [6]. Total number of sprints and markers of acceleration and deceleration capacity are reduced in the last 15 min of the normal duration of a soccer match [7]; therefore, research that targets maintaining these performance outcomes during a soccer match is important.

The glycemic index (GI) differentiates types of carbohydrates based on how fast they cause an increase in blood glucose concentrations [8]. Some studies have indicated consumption of low-GI foods prior to exercise may improve exercise performance compared to high-GI foods [9,10]. Low-GI foods cause a lower insulemic response compared to high-GI foods. Insulin inhibits fat oxidation during exercise [11]; therefore, consumption of low-GI foods might allow increased utilization of fats, lower carbohydrate usage, and preservation of glycogen stores [12,13,14]. Advantages of carbohydrate ingestion with different GIs prior to prolonged endurance exercises are well documented [15,16,17]; however, further studies need to address the possible impact of foods with different GIs on high-intensity intermittent exercise, important for many team sports (e.g., soccer, hockey, rugby). It is important to note that, during low to moderate intensity intervals (e.g., rest and recovery times) of high-intensity intermittent exercise, a considerable amount of energy needed for exercising muscles is provided by fat oxidation [18,19]. Our previous studies have shown some metabolic benefits (i.e., lower insulin levels, higher fat oxidation, lower carbohydrate oxidation and reduced lactate levels) when low-GI meals are consumed before interval treadmill exercise programmed to simulate the repeated high-intensity intervals of a typical soccer match, but performance specific to soccer is difficult to evaluate on a treadmill [20,21,22]. In these previous studies, boiled lentils were compared to high-GI foods (i.e., mashed potatoes with egg whites added to match for protein), but these meals may not be typical before matches for soccer players (or other athletes involved in sports with high-intensity intervals). Endurance athletes often consume sport nutrition bars [23] and surveys of youth soccer players indicate that about 37% consume food, such as sport nutrition bars, up to 1 h before games in an attempt to improve performance [24]; however, the effectiveness of a sport nutrition bar for soccer performance has never been evaluated. From a practical point of view, using sport nutrition bars (high-CHO) can be considered when time is limited before the start of competition. Therefore, the purpose of the current study was to evaluate low- and high-GI sport nutrition bars, consumed before and at half time on metabolism and performance during a soccer-specific field test, which incorporates skills important for soccer performance (i.e., agility running, ball dribbling, kicking accuracy, and ball heading) [25]. We hypothesized that a low-GI sport nutrition bar would be superior to a high-GI sport nutrition bar to improve performance and metabolic responses when consumed before and during a simulated soccer match.

## 2. Materials and Methods 

### 2.1. Participants

Eight male recreational soccer players participated in this study. Their mean ± standard deviation for age, body mass, and predicted maximal oxygen uptake values were 30 ± 7 years, 76.6 ± 8.6 kg, and 56.5 ± 2.5 mL/kg/min, respectively. The University of Saskatchewan Biomedical Research Ethics board approved the study protocol, and all participants signed a consent form before the study began. The approval number is 12–33. The approval date was February 21, 2012.

### 2.2. Study Design

Using a randomized, double blind, counter-balanced cross-over design, low-GI and high-GI sport nutrition bars were consumed two hours before and at half time of a simulated soccer match during which soccer-specific skills (agility, ball dribbling, heading, and kicking accuracy) were assessed. Plasma glucose and insulin, non-esterified free fatty acids (NEFA), as well as fat and carbohydrate oxidation were assessed before and during the simulated soccer match.

### 2.3. Preliminary Test

**Figure 1:**
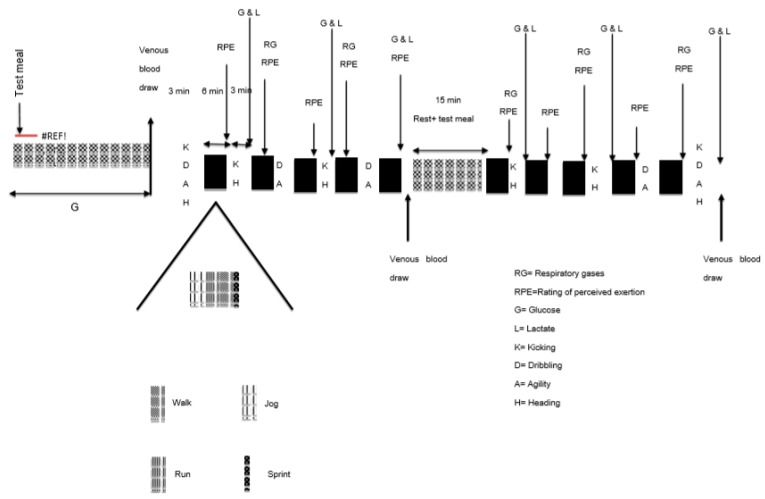
Schematic of the simulated soccer match. “#REF” denotes fingertip blood collection time points for glucose.

**Figure 2:**
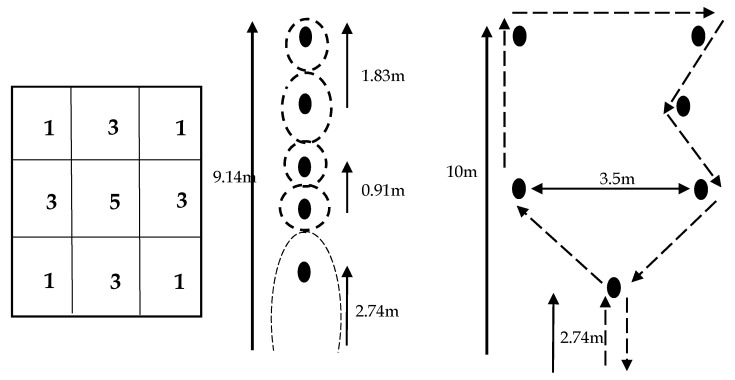
Schematic of the scoring grids for kicking accuracy (**left**), ball-dribbling (**middle**), and agility (**right**) protocols. Arrows represent the distance between the cones. Adopted from Currell et al., [25].

Participants initially had their maximal aerobic power (VO_2max_) estimated by a shuttle-run test that involved running 20 m while the speed increased by 0.14 m/s each minute until volitional exhaustion [26]. The purpose of this test was to determine participants’ aerobic fitness and to determine maximal running velocity, which was used to set speeds during the exercise tests used to evaluate the different feeding conditions. Participants then performed a familiarization test of the simulated soccer match. This test was identical to the test they performed during the sports bar conditions, but was used as a “practice” run. The purpose of this practice run was to minimize any learning effects from one test to another. The practice trial involved performing 10 six-minute sessions of running between two cones that were 20 m apart, where speed was alternated between sprinting, running, jogging, and walking to simulate the exercise performed during an actual soccer match [21,25]. Speeds of walking, jogging, and running were adjusted according to each participant’s predicted maximal aerobic power [26]. The speeds were dictated by “beeps” emitted from a sound system that indicated when the participant was required to reach the next 20 m distance. The speed of walking, jogging, and running were set at 25%, 55%, and 95%, respectively, of the maximal speed reached in the initial maximal shuttle run test [27]. The Bitworks Team Beep Test software (Version 4.1, Bath, UK) was used to write the scripts for each participant. Each 6 min block alternated 60 m of jogging, running, and walking, and 20 m of sprinting. Performance was assessed by tests of either agility running/ball dribbling or kicking/heading of a soccer ball. These pairs of performance tests were alternately performed between the 6 min jogging-running-walking-sprinting sessions with the exception of the first and last 3 min periods of the test during which all four performance tests were completed [25]. The total time of the exercise test (i.e., the 10 six-minute intervals and the testing between intervals) was approximately 90 min which is the same duration as an actual soccer match. The study diagram is shown in Figure 1. This soccer test has been shown to be highly reproducible and is sensitive to improvement with carbohydrate feeding [25]. Separate performance scores were derived for agility, dribbling, kicking accuracy, and heading (Figure 2). Time to complete the agility and ball dribbling courses were recorded for assessment of performance of these tests. The highest vertical jump was recorded for heading performance. A vertical jump measuring device (Vertec, Power Systems (PS), LLC, Knoxville, TN, USA) was used; participants were instructed to use their heads rather than their hands to reach the vanes. Kicking accuracy was scored according to targets set up on a wall net.

### 2.4. Experimental Test 

Participants reported to the lab on two different occasions after a 12 h fast for a low-GI lentil-based bar (Genki Foods Inc., Winnepegosis, MB, Canada) test and a high-GI bar (Clif Bar Inc. Berelely, CA, USA) test. Each condition was separated by at least one week. The GI of the lentil bar was 45 [28] and the GI of the Clif bar was 101 [29]. The crunchy peanut butter flavor Clif bar was used because it most closely matched the Genki Bar for macronutrients and calories. The characteristics of the nutrition bars for a 70 kg participant are shown in Table 1. The testing was double blind, that is, neither the participant nor the researchers knew what type of nutrition bar was consumed. The blinding was achieved by having a separate research assistant prepare the food and having the participant consume the food in an isolated room two hours before the exercise session. Wrappers were removed from the bars and an appropriate amount of bar was placed in plastic bags. The high- and low-glycemic index sport nutrition bars were similar in appearance (i.e., same color and consistency).

On each testing day participants were given enough sports bars to consume 1.5 g/kg body mass available CHO, an amount of carbohydrate that was expected to improve performance when given two hours before high-intensity intermittent exercise [20]. This amount is also within the range of recommended CHO intake prior to endurance exercise performance [8]. Participants also consumed 0.38 g/kg available CHO from the bars at half time of the simulated soccer match. Participants had 20 min to consume the bars before the match and 15 min during half time. Furthermore, the exact amount of water consumption was documented in the first trial and then it was replicated in the second trial to minimize the impact of hydration status. The feeding and the simulated soccer match were separated by two hours. Blood glucose was assessed by fingertip sampling before the food consumption and at 5, 15, 30, 60, 90, and 120 min after consumption. Blood samples from an antecubital vein were taken immediately before, at half time, and after finishing the simulated soccer match for assessment of insulin and non-esterified free fatty acid (NEFA) levels. Fingertip blood samples were collected to assess glucose by using a glucose meter (AccuCheck Compact Plus Sarstedt, Nümbrect, Germany). Venous blood samples were maintained in 10 mL tubes (BD Vacutainer SST) for 30 min to clot. The serum was then separated by centrifugation for 15 min at 3500 rpm and stored at −80 °C. Insulin concentrations were determined using an enzyme-linked immunosorbent assay (ELISA) according to the manufacturer’s directions (STELLUX® Chemi Human Insulin, Alpco Diagnostics, Salem, MA, USA). The serum NEFA assay was performed using a protocol with an oleic acid standard solution as per the manufacturer’s directions (NEFAHR (2), Wako Diagnostics Inc., Richmond, VA, USA). The intra-assay coefficient of variations (CVs) for the insulin, and NEFA assays were <10%. Fingertip blood samples were taken after every second 6 min exercise interval during the simulated soccer match to measure glucose and lactate levels. Blood lactate measurement was assessed using BM-Lactate test strips and the Accutrend® Lactate analyzer (Roche Group; Mannheim, Germany). The K4 b2® (Cosmed USA, Chicago, IL, USA), a portable gas exchange system was used to measure oxygen consumption (VO_2_), and carbon dioxide output (VCO_2_). Respiratory gases were collected during every second 6 min exercise interval to estimate carbohydrate and fat oxidation. Carbohydrate and fat oxidation rates were estimated from VO_2_ and VCO_2_ by using stoichiometric equations [30]. Rating of perceived exertion, using the modified 10-point Borg scale, was collected after each 6 min interval [31].

### 2.5. Dietary and Physical Activity Monitoring

Participants recorded their dietary intake and physical activity for the 24 h before the feeding conditions. These were photocopied and given back to the participants so they could duplicate their diets and physical activity levels during subsequent feeding conditions. This ensured that participants arrived for each exercise feeding condition with similar diets and exercise the previous 24 h.

### 2.6. Statistical Analysis

All variables were analyzed with a two-factor repeated measures analysis of variance (ANOVA) with factors for food condition (low-GI lentil bar vs. high-GI Clif Bar) and time during the exercise test. When there was a time main effect or an interaction between condition and time, a Least Significant Difference (LSD) post-hoc test was used to determine differences between pairs of means. All variables were also assessed for order effects with a two-factor ANOVA with factors for order (first condition vs. second condition) and time during the exercise test. Significance was accepted at a *p*-value less than 0.05. All results are reported as means and standard deviations.

## 3. Results

### 3.1. Blinding, Order Effects, and Adverse Events

When queried as to which bar condition participants thought they had consumed, two participants guessed correctly, while all others responded that they were unsure; this indicated the success of the blinding. There were no order effects or order*time interactions for any of the outcome variables (*p* > 0.05). There were no adverse events associated with the study. None of the participants complained of gastrointestinal discomfort after consumption of the bars.

### 3.2. Glucose and Insulin Responses 

**Figure 3:**
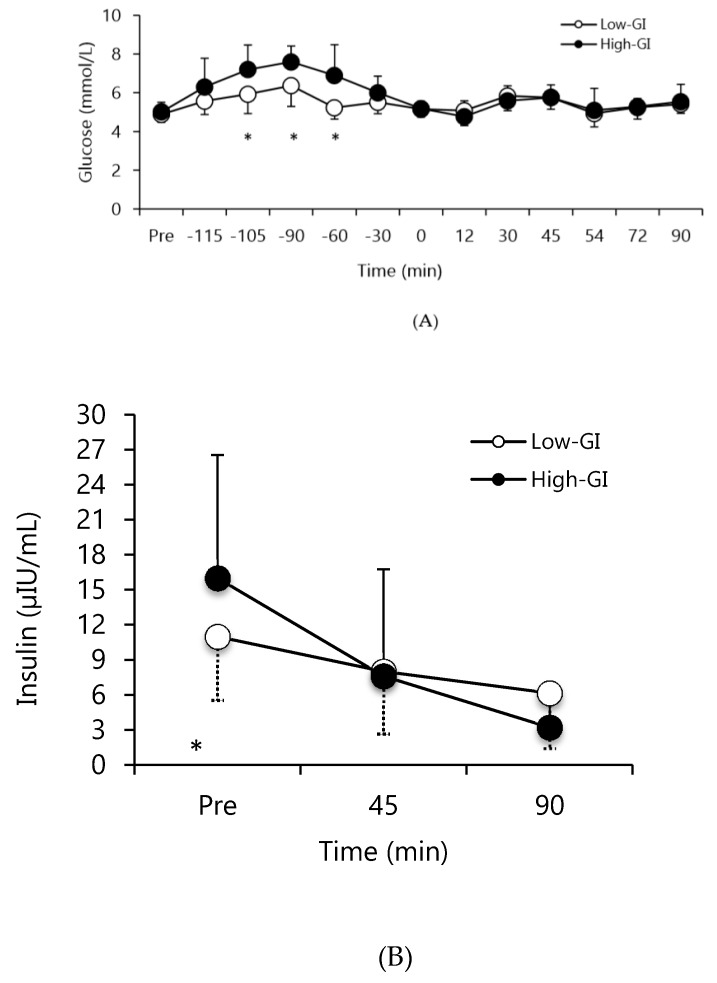
(**A**) Plasma glucose concentrations before and during the simulated soccer match, (**B**) Insulin concentration before, at half time, and at the end of the simulated soccer match (* *p* < 0.05 low-GI vs. high-GI sport nutrition bar). “Pre” denotes fingertip blood sample collection prior to consumption of the sport nutrition bars.

There was a condition*time interaction for glucose and insulin. The high-GI condition resulted in higher glucose concentrations than the low-GI condition at 105, 90, and 60 min before the simulated soccer match (*p* < 0.05; Figure 3A). The insulin response was higher in the high-GI condition compared to the low-GI condition at two hours after bar consumption (*p* < 0.05; Figure 3B).

### 3.3. Serum NEFA and Substrate Oxidation

**Figure 4:**
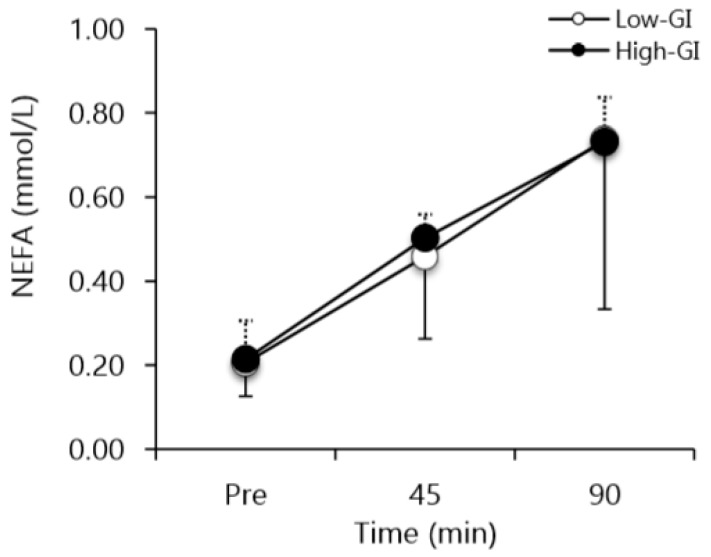
Non-esterified fatty acid concentrations before, at half time, and at the end of the simulated soccer match for low- and high-GI conditions. Values are means ± standard deviation (SD). Time main effect (*p* < 0.05) with 90 min > 45 min > pre (*p* < 0.05). “Pre” denotes venous blood draw prior to the simulated soccer match.

**Figure 5:**
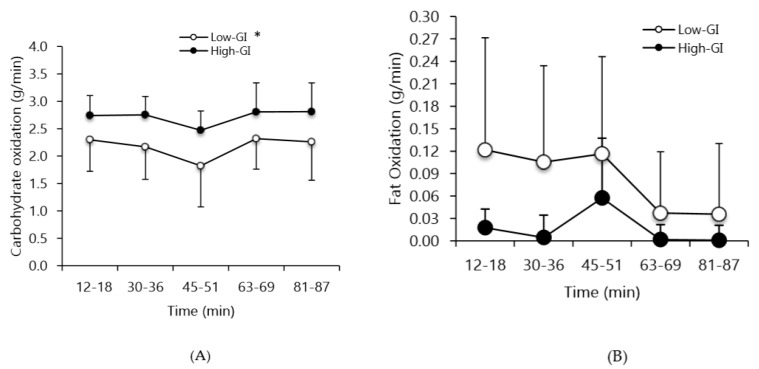
(**A**) Rate of carbohydrate oxidation, (**B**) and fat oxidation during the simulated soccer match in the low glycemic index and high glycemic index conditions. Values are means and SD. * Main effect for condition for carbohydrate oxidation, with the low-GI condition lower than the high-GI condition; *p* < 0.05. There was a time main effect (*p* < 0.05) for fat oxidation (45–51 min > 63–69 min, 81–87 min; *p* < 0.05).

**Figure 6:**
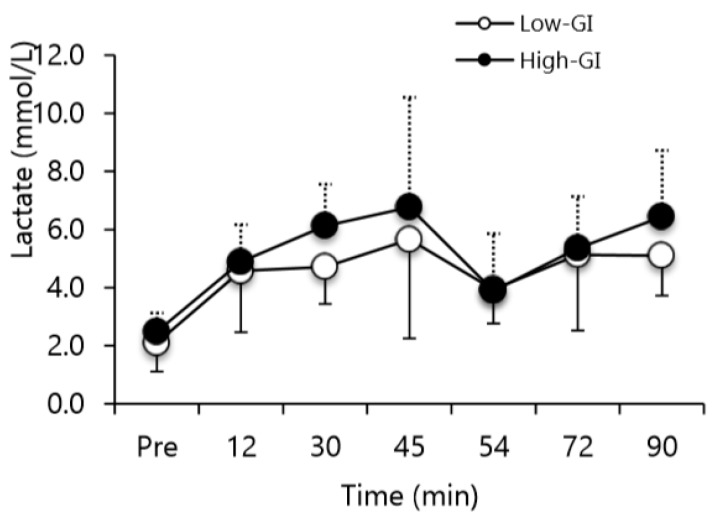
Lactate concentrations before and during the simulated soccer match between low- and high-glycemic index conditions. Values are means ± SD. Time main effect (*p* < 0.05) with all values except 54 min >pre, and 45 min, 90 min >54 min (*p* < 0.05).

NEFA concentration was not different prior to the exercise test in low-GI vs. high-GI conditions (Figure 4). NEFA concentrations significantly increased in the low-GI and high-GI conditions at 45 min and 90 min of exercise (time main effect, *p* < 0.05; Figure 4). No significant difference was seen between the conditions. During the low-GI condition, carbohydrate oxidation was significantly lower compared to the high-GI condition (*p* < 0.05; Figure 5A). There was no difference between conditions for fat oxidation (*p* = 0.14; Figure 5B). There was a time main effect (*p* < 0.05) for fat oxidation with higher rates at 45–51 min versus 63–69 min and 81–87 min (*p* < 0.05). No significant difference was observed for lactate concentrations between the two conditions (Figure 6). There was a time main effect (*p* < 0.01) for lactate, with values increasing at all time-points, except at 54 min (i.e., after half time) compared to baseline (*p* < 0.05). The lactate at 54 min was lower than at 45 min and 90 min (*p* < 0.05).

### 3.4. Skill Performance and Rating of Perceived Exertion

There were condition*time interactions for the agility and heading tests (*p* < 0.05). A significant improvement on the agility test and vertical jump height during simulated heading late in the soccer match (72 min) was observed after consuming the low-GI versus high-GI bar (Table 2; *p* < 0.01). No differences were apparent between bar conditions for skills performance of ball dribbling or kicking accuracy (Table 2). There was a time main effect for rating of perceived exertion (RPE; increasing throughout the simulated soccer match; *p* < 0.05); however, no significant difference was observed between the two conditions (mean RPE throughout the 90 min soccer match: low-GI = 5.2 ± 1.5 versus high-GI = 5.4 ± 1.3; *p* > 0.05).

## 4. Discussion

The main finding of this study was that a low-GI sport nutrition bar consumed two hours before and at half time during a simulated soccer match elicited lower carbohydrate oxidation throughout the match and improvements in agility performance and heading (i.e., vertical jump height) late in the match compared to a high-GI sport nutrition bar. In line with this potential for CHO supplementation to improve performance, in a systematic review, Russell and Kingsley stated that six out of eight included studies found that CHO ingestion in the form of 6%–8% solution of glucose, sucrose, or maltodextrin (which would have a high-GI; i.e., GI > 70 [8]) was linked with an improvement of at least one aspect of soccer skill performance [32]. However, to the best of our knowledge this is the first study to address the influence of low- and high-GI sport nutrition bars consumed shortly prior to prolonged, high-intensity, intermittent exercise, which is typical for many team sports. Although we found improvements in some performance measures with the low-GI sport nutrition bar condition at the 72 min time point, this did not persist to the 90 min time point of the simulated soccer match (Table 2). This may be due to lack of adequate statistical power, or perhaps the GI of the bar consumed does not make a difference this late in the match (i.e., glycogen depletion may be at a low enough level in both conditions to impair performance).

Our metabolic findings are in agreement with our previous work with soccer players. In our previous work, we showed that low-GI foods (i.e., lentils; with GI ranging from 29–36; where low-GI is defined as < 55 [8]) consumed before a simulated soccer match on a treadmill reduced carbohydrate oxidation [20], increased fat oxidation [21], and tended to reduce glycogen usage [22] compared to conditions where high-GI foods (i.e., instant mashed potatoes, white bread, and egg whites to match for protein; GI ranging from 75–81; where high-GI is defined as > 70 [8]) were consumed before exercise. In these previous studies we did not see any difference between low-GI and high-GI conditions when performance was evaluated by repeated sprints at the end of the simulated treadmill test. The current study used skill performance that was quite different from these previous studies and more specific to soccer performance. The current study also used a dietary condition (i.e., sports nutrition bars) which is more likely to be used by soccer players before matches when less time is available for food consumption [5].

In line with our previous studies [20,22] glucose concentrations were significantly higher in the high-GI condition in the first 60 min following consumption versus the low-GI condition. Consequently, insulin response in the high-GI condition was higher than the low-GI condition, which might explain the higher carbohydrate oxidation during the test in the high-GI condition. Insulin inhibits fat oxidation, necessitating greater carbohydrate oxidation and potentially greater glycogen usage [11]. Muscle glycogen is a major substrate during prolonged intermittent high-intensity exercise to provide high rate of ATP re-synthesis [3,4,33]. In this study, the carbohydrate oxidation rate was lower in the low-GI condition compared to the high-GI condition averaged across all time points (i.e., there was a “condition” main effect), and the potential for glycogen sparing might have contributed to improved exercise performance (i.e., agility running and heading) late in the simulated soccer match. In line with this, Saltin [6] showed that total walking distance and sprinting speed were reduced in soccer players with lower glycogen content versus those with higher glycogen content late in a soccer match. Bendiksen et al. [34] reported that utilization of muscle glycogen was significantly lower in the last 30 min of a match suggesting an important role of sustained availability of glucose later in the match. It should be noted, however, that we did not directly assess muscle glycogen in the current study; therefore, we cannot make conclusions on whether there was sparing of glycogen during the low-GI condition. 

It has been postulated that a reduction in lipolysis rate, and therefore NEFA, will occur following high-GI pre-exercise meals [15,35]; however, we found no significant difference between low-GI and high-GI conditions for appearance of NEFA in the blood. This discrepancy might have been related to the greater intensity of our test protocol versus the other studies. During higher intensity exercise, plasma NEFA concentrations decrease while glucose and glycogen utilization increase in skeletal muscle [36]. A limited rate of fat oxidation is thought to be connected with a lower flux of long chain NEFA across the mitochondrial membrane [37,38]. An alternative explanation to the lack of difference in NEFA concentration between conditions may be that the high-GI condition resulted in lower intramyocellular lipids utilization rather than lower lipolysis from adipose tissue. 

The main limitation of our study was the assumption that reduced carbohydrate oxidation would lead to sparing of muscle glycogen. Direct analysis for glycogen levels by muscle biopsy would strengthen future studies comparing high- versus low-GI foods. We attempted to standardize glycogen levels between trials by having participants match their dietary intake and physical activity levels the day before each trial. For better control, it would be preferable to provide standardized meals to participants the day before trials. We would expect an increase in NEFA release from adipose tissue with the lower insulin concentration in the low-GI condition, but this was not observed. A limitation is that we did not assess glycerol, which may give more precise data on lipolysis. Another limitation is that we tested athletes after an overnight fast. The typical practice of a soccer player would most likely be to have a small breakfast and then consume a small amount of carbohydrate before the soccer match. We supplied enough of the bars to provide 1.5 g/kg available carbohydrate before the soccer match, an amount of carbohydrate that is recommended for improvement in endurance performance [8]. This required consumption of approximately five bars by each participant which totaled approximately 760 kcal (Table 1). We felt the addition of a breakfast before the bar consumption would result in excess fullness in participants. Additional limitations include a relatively small participant number, and the fact that our soccer match was simulated, rather than being an actual soccer match. Future studies could focus on the effects of consuming the bars before actual soccer matches.

## 5. Conclusions and Practical Application

Previous studies carried out in our lab using treadmill protocols to measure soccer performance (i.e., 1 min intervals of high-intensity running at the end of a simulated soccer match) were not very specific to soccer performance. This motivated the use of a field simulated soccer test incorporating soccer skills (i.e., agility, dribbling, kicking, and heading) to optimize the specificity of the test to the sport of soccer. Another novel aspect of the study was the assessment of sport nutrition bars given with adequate amount of recommended available carbohydrates (i.e., 1.5 g/kg) before endurance exercise performance. Previous research has generally shown that consumption of sports nutrition bars has no effect on endurance exercise performance [39,40,41]; however, these studies evaluated the effect of only a single sports nutrition bar before exercise. This would deliver well below the recommended amount of available carbohydrate for improving exercise performance; therefore, consumption of higher number of sports nutrition bars might seem practical. Sport nutrition bars containing high-CHO can act as an immediate snack, in particular, when soccer players are under time constraints before matches.

In conclusion, a low-GI sport nutrition bar consumed before a simulated soccer match elicited a lower carbohydrate oxidation rate and a modest improvement in performance (i.e., better agility and heading performance late in a simulated soccer match) versus a high-GI sport nutrition bar. Further studies are required to investigate how sport nutrition bars varying in GIs could impact soccer skill performance during prolonged match play (i.e., over/extra time, penalty kicks) when carbohydrate stores will be further depleted.

## Figures and Tables

**Table 1:** Characteristics of the sport nutrition bars for a 70 kg participant.

Description	Low-GI	High-GI
Energy (kcal)	758	761
Fat (g)	19	20
Total Carbohydrate (g)	127	116
Available carbohydrate (g), i.e., Total carbohydrate minus fiber	105	105
Protein (g)	39	31
Glycemic index	45	101

GI, glycemic index.

**Table 2:** Performance variables during the simulated soccer match.

Condition	Low-GI							High-GI					
Time (Min)	12	30	45	54	72	90	12		30	45	54	72	90
Ball Dribbling (s)	14.0 ± 2.6	12.8 ± 2.3	11.8 ± 2.0	12.2 ± 2.2	12.6 ± 1.9	12.3 ± 2.1	13.8 ± 2.6		13.1 ± 2.3	12.1 ± 2.2	12.6 ± 1.9	12.1 ± 2.4	12.7 ± 2.7
Heading (cm)	20.5 ± 6.5	24.4 ± 4.1	24.1 ± 4.4	23.3 ± 3.5	24.7 ± 4.3 *	23.3 ± 0.9	20.3 ± 7.9		23.8 ± 4.8	23.5 ± 5.2	22.5 ± 5.2	22.2 ± 4.9	22.5 ± 5.1
Kicking (arbitrary units)	10.0 ± 5.7	11.8 ± 3.0	11.1 ± 5.5	13.1 ± 7.1	12.4 ± 4.0	9.1 ± 4.3	10.8 ± 6.5		10.1 ± 5.4	9.3 ± 5.9	9.0 ± 2.3	12.1 ± 4.5	12.8 ± 6.2
Agility (s)	6.1 ± 0.4	6.0 ± 0.6	6.0 ± 0.6	5.9 ± 0.5	5.7 ± 0.4 *	6.0 ± 0.6	6.1 ± 0.4		6.1 ± 0.6	5.9 ± 0.6	6.1 ± 0.5	6.1 ± 0.6	5.8 ± 0.6

Values are means and standard deviation (SD). * Significantly different in the low-GI versus high-GI condition (*p* < 0.01).
